# A coastal coccolithophore maintains pH homeostasis and switches carbon sources in response to ocean acidification

**DOI:** 10.1038/s41467-018-04463-7

**Published:** 2018-07-20

**Authors:** Yi-Wei Liu, Robert A. Eagle, Sarah M. Aciego, Rosaleen E. Gilmore, Justin B. Ries

**Affiliations:** 1 Department of Earth and Environmental Sciences, University of Michigan, 2534 C. C. Little Building, 1100 North University Avenue, Ann Arbor, MI 48109 USA; 2 Université de Brest, UBO, CNRS, IRD, Ifremer, Institut Universitaire Européen de la Mer, LEMAR, Rue Dumont d′Urville, 29280 Plouzané, France; 3 Institute of Earth Sciences, Academia Sinica, 128, Sec. 2, Academia Road, Nangang, 11529 Taipei Taiwan; 4 Institute of the Environment and Sustainability, University of California – Los Angeles, La Kretz Hall, 619 Charles E. Young Dr. E #300, Los Angeles, CA 90024 USA; 5 Department of Atmospheric and Oceanic Sciences, University of California – Los Angeles, Math Sciences Building, 520 Portola Plaza, Los Angeles, CA 90095 USA; 6 Department of Geology and Geophysics, University of Wyoming, 1000 E. University Avenue, Laramie, WY 82071-2000 USA; 7 Department of Marine and Environmental Sciences, Marine Science Center, Northeastern University, 430 Nahant Road, Nahant, MA 01908 USA

## Abstract

Ocean acidification will potentially inhibit calcification by marine organisms; however, the response of the most prolific ocean calcifiers, coccolithophores, to this perturbation remains under characterized. Here we report novel chemical constraints on the response of the widespread coccolithophore species *Ochrosphaera neapolitana* (*O. neapolitana*) to changing-CO_2_ conditions. We cultured this algae under three *p*CO_2_-controlled seawater pH conditions (8.05, 8.22, and 8.33). Boron isotopes within the algae’s extracellular calcite plates show that this species maintains a constant pH at the calcification site, regardless of CO_2_-induced changes in pH of the surrounding seawater. Carbon and oxygen isotopes in the algae’s calcite plates and carbon isotopes in the algae’s organic matter suggest that *O. neapolitana* utilize carbon from a single internal dissolved inorganic carbon (DIC) pool for both calcification and photosynthesis, and that a greater proportion of dissolved CO_2_ relative to HCO_3_^−^ enters the internal DIC pool under acidified conditions. These two observations may explain how *O. neapolitana* continues calcifying and photosynthesizing at a constant rate under different atmospheric-*p*CO_2_ conditions.

## Abstract (web summary)

Calcifying species have diverse responses to ocean acidification, but the underlying mechanisms are not well-constrained. Here, Liu et al. show that *O. neapolitana* maintains its calcification site pH and utilizes more CO_2_ compared to HCO_3_^−^ to support its growth under high-CO_2_ conditions.

## Introduction

CO_2_-induced ocean acidification (OA) is reducing the calcium carbonate saturation state of seawater, potentially making it harder for calcifying marine organisms to build their shells and skeletons^1–3^. However, controlled laboratory experiments suggest that organisms have extremely varied calcification responses to CO_2_-induced OA, with some species exhibiting resilience or even showing positive calcification responses to high-CO_2_ conditions^4–11^. It has been hypothesized that such diverse responses to CO_2_-induced OA may result from differing capacities of marine calcifying organisms to actively remove protons, H^+^, from their calcifying medium, thereby raising the carbonate ion concentration, [CO_3_^2−^], and calcium carbonate saturation state of the calcifying medium relative to seawater^12–15^.

In addition to regulating calcification site pH via proton pumping, organisms that perform both photosynthesis and calcification may also influence carbonate chemistry at the site of calcification via photosynthetic drawdown of dissolved inorganic carbon (DIC), DIC release via respiration, and/or proton release via calcification (i.e., Ca^2+^ + HCO_3_^−^ → H^+^ + CaCO_3_).

Coccolithophores are unicellular flagellate algae with the ability to produce calcite plates (coccoliths) within vesicles that they assemble in an interlocking fashion on the exterior surface of their cell wall. Coccolithophores inhabit tropical to subpolar environments throughout the ocean^16,17^. They are the most prolific producers of CaCO_3_ in the ocean, accounting for nearly half of global production^18,19^. Therefore, the coccolithophore response to elevated *p*CO_2_ is important from both ecological and global biogeochemical perspectives.

A recent study suggested that, on geological timescales, the combination of elevated dissolved CO_2_ and elevated total alkalinity (TA) supports the production of thicker coccoliths^20^. However, controlled laboratory experiments suggest that the coccolithophore calcification and photosynthesis response to elevated *p*CO_2_ is variable and complex, with disparate results observed amongst different species and even different strains of the same species^5,9,21,22^. It is possible that species- and strain-specific differences in coccolithophores’ ability to regulate pH and carbonate chemistry at their site of calcification contributes to the variability in their responses to OA. However, few measurements of pH at the site of calcification within coccolithophores have been reported^23^, with none addressing the impact of elevated *p*CO_2_ on calcification site pH.

Boron isotope ratios (*δ*^11^B) in biogenic carbonates are used as a proxy for seawater pH, based on the observation that seawater pH controls the *δ*^11^B composition of dissolved borate, which is assumed to be the primary boron species incorporated into abiogenic and biogenic carbonates^24^. However, discrepancies observed between carbonate *δ*^11^B-derived pH and known seawater pH indicate that *δ*^11^B compositions of biogenic carbonates may represent pH at the site of calcification, which may reflect biological control of pH at the site of calcification in some species, or seawater pH in other species, or some combination of seawater pH and biological control. This use of *δ*^11^B as a proxy of calcification site pH has been most widely applied to scleractinian corals^12,13,15,25,26^, with only one study to date applying this approach to a species of coccolithophore (*Emiliania huxleyi*)^27^.

Previous studies have used fluorescent dyes to estimate cytosolic and coccolith vesicle pH, and their sensitivity to changes in external seawater pH^23,28^. However, our approach adds new information because the coccolith *δ*^11^B should reflect the pH of the coccolith-forming vesicle at the precise time and location of coccolith formation, rather than vesicle pH at the time of dye injection and imaging. Here, we also consider stable carbon isotope data from inorganic and organic portions of coccolithophores to gain insight into how coccolithophores utilize different species of DIC for both photosynthesis and calcification, since elevated atmospheric-*p*CO_2_ will change the distribution of DIC species in seawater.

*Ochrosphaera neapolitana (O. neapolitana)* is a mostly non-motile coccolithophore species that is widely distributed along the coastal zone of the North Atlantic and Indian Oceans, Mediterranean Sea, and Japanese Current region^29–31^. This species has a unique mechanism for producing coccoliths, wherein the coccolith vesicle is formed at the periphery of the cell instead of adjacent to the nuclear membrane, as for *Emiliania huxleyi (E. huxleyi)*, or between the nucleus and plasma membrane, as for *Pleurochrysis carterae*^29^. Unlike the coccolithophore species *E. huxleyi* and genera *Gephyrocapsa*, *Coccolithus*, and *Calcidiscus*, which produce highly calcified placoliths, i.e., lith-structures with two or more well-developed shields, *O. neapolitana* produces more lightly calcified muroliths, i.e., lith-structures without well-developed shields^29,32,33^. The production of less calcified liths by *O. neapolitana* could be linked to a different strategy for allocating dissolved inorganic carbon resources under different environmental conditions.

Furthermore, it has been shown that *O. neapolitana* precipitates high-magnesium calcite, while the well-studied coccolithophore genera *Emiliania* and *Gephyrocapsa* produce low-magnesium calcite liths, providing further evidence that *O. neapolitana* calcifies in a different manner than other species of coccolithophores^34^. However, few studies have explored the allocation of dissolved inorganic carbon resources between photosynthesis and calcification within *O. neapolitana* (e.g., ref.^35^). For these reasons, along with the logistical need to culture large volumes of coccolithophores for measuring isotope ratios of low-abundance elements such as boron, the relatively fast-growing species *O. neapolitana* was used to develop our combined *δ*^11^B and *δ*^13^C approach for constraining the response of calcifying fluid chemistry in coccolithophores to OA.

Here, we report on controlled laboratory experiments investigating the impact of CO_2_-induced OA on the calcifying fluid dynamics and utilization of DIC within the coastal coccolithophore species *O. neapolitana*. The effective *p*CO_2_ range examined spans 226 ± 11 µatm (glacial) to 521 ± 19 µatm (year 2070). We make inferences about this speciesʼ (1) ability to regulate calcifying fluid pH using boron isotopes as a proxy of calcifying fluid pH, (2) utilization of carbon species in calcification and photosynthesis, using stable isotopes of carbon and oxygen, and (3) photosynthetic and calcification responses to CO_2_-induced OA, using their PIC/POC ratio. Our results suggest that *O. neapolitana* maintains a constant pH at the site of calcification and can maintain relatively constant calcification-to-photosynthesis ratios across a range of seawater pH conditions by utilizing higher proportions of CO_2_ compared to HCO_3_^−^ as seawater pH decreases.

## Results and Discussion

### Uptake of dissolved boron species by coccolithophores

**Fig. 1:**
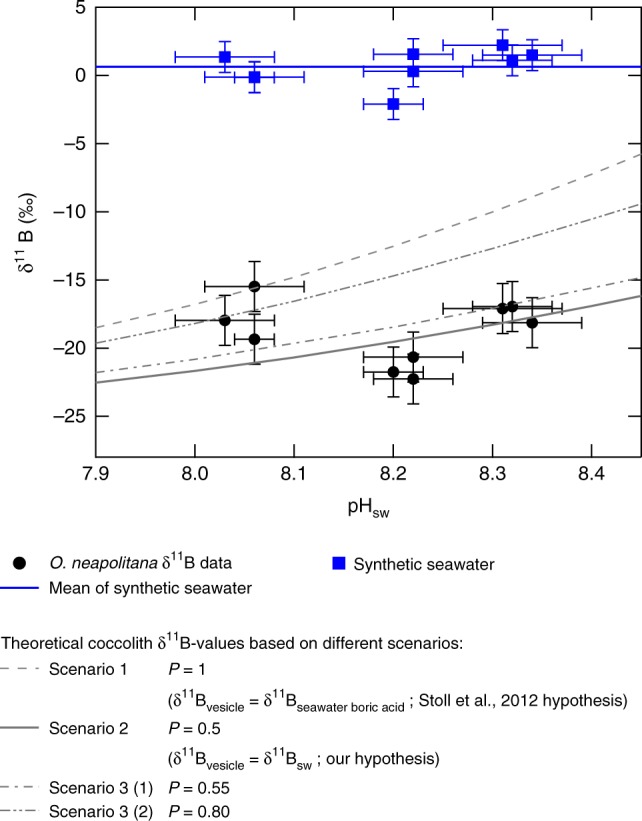
Impact of transporting different boron species to calcification site on coccolith boron isotopic composition. The relationship between *δ*^11^B and pH_SW_ for three B-transport scenarios (gray dash line, gray solid line, gray dash-dot line, and gray dash-double dots line for scenarios 1, 2, 3(1), and 3(2), respectively) are compared to *δ*^11^B-pH_SW_ relationships for coccoliths from the present study (solid black circles). The B-transport scenarios were calculated relative to average *δ*^11^B of culture seawater (blue line is average *δ*^11^B of culture seawater across treatments). *P* is defined as the proportion of total boron entering the calcifying vesicle as boric acid. Individual culture seawater *δ*^11^B results are shown as solid blue squares. Although the different B-transport scenarios assume different proportions of seawater boric acid and borate are transported to the coccolith calcifying vesicle, all scenarios assume that the B species will be redistributed in the vesicle pursuant to vesicle pH. The models also assume that only borate ion is incorporated into the coccolith calcite. Scenario 2, boric acid and borate enter calcifying vesicle in proportions that exist in seawater, such that *δ*^11^B_vesicle_ = ^11^B_sw_, best describes the *δ*^11^B-pH_SW_ relationships for coccoliths from the present study (gray solid line). Deviations from the scenario 2 curve occur under the most acidified treatment, which may reflect biological elevation of pH at the site of calcification. Error bars are 2 SD for *y*-axis and 1 SD for *x*-axis. See detailed calculation in Supplementary Note 1

The coccolith *δ*^11^B values, fractionated from the culture medium *δ*^11^B of 0.64‰, range from −15‰ to −25‰ (Fig. 1). Although boron isotopic compositions of marine carbonates, such as corals and foraminifera, have been widely studied and applied as proxies for seawater or calcification site pH (e.g., refs.^12,15,25,36,37^), the *δ*^11^B composition of coccolithophore calcite has not been well-constrained. Non-charged boric acid has been shown to travel across the cell membranes of plants via diffusion, a process that discriminates against the positively charged borate ion^38,39^. Therefore, Stoll et al.^40^ hypothesized that boric acid enters coccolithophore cells exclusively through a similar mechanism, given the relatively high concentration of boron in seawater compared to freshwater. In contrast, the boron isotopic composition of many marine biogenic carbonate archives has been found to reflect calcification site pH^12,13,15,25,26^, supporting the hypothesis that *δ*^11^B of total boron within the parent fluids of calcification is identical to that of seawater, and that only borate ion is incorporated in these carbonates. Because questions remain as to the source(s) and pathway(s) of inorganic boron into coccolithophores, we discuss the compatibility of the results of the present study with three potential boron-uptake scenarios: (1) only boric acid can penetrate the coccolithophore cell membrane to the calcification site, yielding total boron *δ*^11^B at the calcification site highly enriched relative to ambient seawater; (2) boric acid and borate penetrate the cell membrane in proportions equivalent to their abundance in seawater, yielding total boron *δ*^11^B at the calcification site identical to ambient seawater; and (3) an intermediate scenario in which both seawater boric acid and borate ion penetrate the cell membrane to the site of calcification, but with higher boric acid:borate ratios than in the ambient seawater—thereby yielding total boron *δ*^11^B at the calcification site slightly enriched relative to ambient seawater.

In the first scenario, if only boric acid enters the coccolithophore cell, *δ*^11^B of total B at the calcification site will be about 5–10‰ higher than that of the culture seawater across the range of pH investigated in this study. Thus, assuming that no further fractionation of boron isotopes occurs throughout the calcification process and only borate ion is incorporated into coccolith calcite, the coccolith *δ*^11^B value should also be about 5–10‰ higher than the theoretical borate curve for seawater (Fig. 1, gray dash line). In the second scenario, where boric acid and borate penetrate the cell membrane in proportions equivalent to their relative abundance in seawater, coccolith *δ*^11^B value should follow the gray solid line in Fig. 1. Finally, for the third scenario (e.g., Fig. 1, gray dash-dot line and gray dash-double dots line), coccolith *δ*^11^B value should fall between the gray dashed line and gray solid line in Fig. 1.

In the first scenario, the predicted borate *δ*^11^B values (Fig. 1, gray dash line) are higher than most coccolith *δ*^11^B measurements. If this was driven purely by calcification site pH, the values could be explained by *O. neapolitana* downregulating their calcification site pH to <7.9, which would reduce the calcite saturation state and be relatively unfavorable for calcification.

**Fig. 2:**
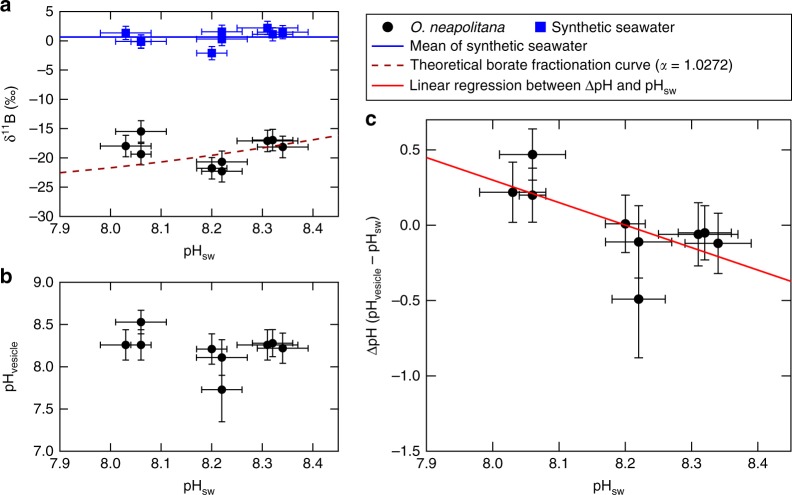
Seawater pH vs. coccolith *δ*^11^B and boron-isotope-inferred dynamics of calcifying vesicle pH. **a** Seawater pH vs. *δ*^11^B of coccolith calcite (solid black circles), *δ*^11^B of culture seawater (solid blue squares; blue line is average *δ*^11^B across treatments), and theoretical *δ*^11^B of seawater borate (brown dash curve). If total boron *δ*^11^B and pH of the seawater and calcifying vesicle are identical, and only borate is incorporated into the coccolith calcite, coccolith *δ*^11^B results should lie on the theoretical *δ*^11^B-pH_SW_ curve—which is not the case. Deviation from this relationship under the most acidified treatment is interpreted as reflecting biological elevation of pH at the site of calcification. **b** Seawater pH vs. coccolith vesicle pH inferred from *δ*^11^B of coccolith calcite, and temperature, salinity, and *δ*^11^B of seawater. **c** Seawater pH vs. offset between coccolith calcification site pH and culture seawater pH (∆pH). The linear regression between ∆pH and seawater pH (pH_sw_) is shown in red. Error bars are 2 SD for *y*-axis and 1 SD for *x*-axis

In the second scenario, the predicted borate *δ*^11^B values closely match the coccolith *δ*^11^B measurements, especially for pH above 8.15. Only under lower pH (pH = 8.05) do the coccolith *δ*^11^B values deviate positively from the predicted borate *δ*^11^B values (Fig. 2, gray solid line). This positive deviation can be explained by the maintenance of a constant and elevated pH in the calcification vesicle relative to seawater pH, which has been observed in many other marine calcifying organisms (e.g., refs.^15,41,42^). This scenario has been widely adopted by researchers using *δ*^11^B of biogenic carbonates as a proxy for calcification site pH in marine calcifying organisms^12,13,15,25,26^.

Alternatively, both boric acid and borate may be transported into the coccolith vesicle (scenario 3) but with the boric acid:borate ratio elevated compared to seawater. This latter scenario should be considered the least likely as the authors are aware of no mechanism for the partial discrimination against borate during boron uptake to the site of calcification and, if accurate, would constitute a hitherto unrecognized mechanism for boron transport in coccolithophores (see further discussion in Supplementary Note 1).

The scenario wherein the total boron *δ*^11^B in the coccolith vesicle is equivalent to the total boron *δ*^11^B in seawater (scenario 2) is most compatible with the results of the present study. This suggests that the active biological elevation of coccolith vesicle pH drives the offset in coccolith *δ*^11^B from the theoretical borate *δ*^11^B-pH curve, which is apparent in the lowest pH treatment.

### Coccolith *δ*^11^B as an indicator of calcifying vesicle pH

Coccolith δ^11^B was measured as a proxy for calcifying vesicle fluid pH in cultures bubbled with CO_2_-gas mixtures formulated at 280, 400, and 750 p.p.m., which resulted in average seawater pH values of 8.33, 8.22, and 8.05 in cultures. Based on calculations using the instrumental measurement of DIC and total alkalinity in the experimental treatments, the algal cultures were subjected to *p*CO_2_ levels of 226, 339, and 521 µatm for the three treatments, with the difference between the nominal and effective *p*CO_2_ arising from incomplete mixing between the introduced gases and culture solutions and drawdown of aqueous CO_2_ by the coccolithophores. The average *δ*^11^B of the total dissolved boron in the synthetic culture medium from individual tanks was 0.64 ± 1.30‰ (1*σ*, *n* = 9), about 39‰ lower than natural seawater. Notably, the *δ*^11^B of both boric acid (B(OH)_3_), dominating at low pH, and borate ion (B(OH)_4_^−^), dominating at high pH, are fractionated relative to *δ*^11^B of total seawater boron as a function of solution pH^24^, with *δ*^11^B of dissolved B(OH)_4_^−^ increasing with increasing seawater pH. Because only the B(OH)_4_^−^ form of boron is thought to be incorporated into the calcite lattice (see discussion above)^24,40^, *δ*^11^B of coccolith calcite should represent *δ*^11^B of dissolved B(OH)_4_^−^ in the calcifying vesicle fluid. Assuming that *δ*^11^B of total boron in the calcifying vesicle is inherited from the culture solution, then calcifying vesicle fluid pH can be estimated from the fractionation between *δ*^11^B of total boron in the culture medium and *δ*^11^B of coccolith calcite.

The observed difference in *δ*^11^B between coccolith calcite, ranging from −15‰ to −25‰, and culture medium, 0.64‰, yields a calcifying vesicle fluid pH between 7.7 and 8.5, with a mean pH of 8.2, based on calculations using the most widely applied boron fractionation factor^43^ (Fig. 2a, b).

In a previous study, Anning et al.^23^ used pH-sensitive fluorescent dyes to detect coccolith vesicle pH. They observed a bimodal distribution of calcifying vesicle pH within the population of cultured cells: one spanning pH 6.8–7.2 and the other spanning pH 7.6–8.3^23^. Although our study examines a different species of coccolithophore, the higher pH mode in the previous study^23^ is consistent with vesicle pH estimated from *δ*^11^B data in the present study. Thus, the higher pH mode reported in the pH-sensitive dye study, which is consistent with vesicle fluid pH estimated in the present study from coccolith *δ*^11^B and thus representative of vesicle pH at the precise time and location of lith formation, may represent pH of vesicles that are actively engaged in lith production. In contrast, the lower pH mode may reflect pH of vesicles that are not actively forming liths.

Notably, no statistically significant (*p* > 0.05) correlation was observed between coccolith *δ*^11^B and ambient seawater pH, which is consistent with *δ*^11^B-based observations of calcifying vesicle pH for the coccolithophore *E. huxleyi*^27^. Instead, a significant negative trend was observed in the present study between seawater pH (pH_sw_) and the offset (∆pH) between vesicle pH (pH_vesicle_) and pH_sw_ (*R*^2^ = 0.46, *p* < 0.05) (Fig. 2c). Together, these analyses suggest that vesicle pH is insensitive to changing pH_sw_, and that increasing ∆pH with decreasing pH_sw_ reflects the algae’s compensatory response to OA, which maintains vesicle pH and carbonate chemistry favorable for coccolith formation even under increasingly acidic conditions. This response is distinct from the many types of calcifying marine organisms, including corals, foraminifera, and coralline algae, which show a clear decline in calcification site pH with decreasing seawater pH^12–14,41,44^. Therefore, these results suggest that this species of coccolithophore exerts an unusual degree of control over its calcification site pH.

It has been suggested that photosynthesis can benefit calcification in several ways, including providing energy for proton pumping or other enzyme-driven ion exchange and generating hydroxyl ions that neutralize protons released via calcification^45^, thereby increasing calcium carbonate saturation state at the site of calcification. These mechanisms may assist coccolithophores in maintaining constant calcification site pH as ambient seawater pH declines, but only with commensurate increases in rates of photosynthesis, which is potentially supported by higher seawater DIC levels associated with elevated *p*CO_2_^5^.

Alternatively, calcification can also benefit photosynthesis by generating protons that increase seawater concentrations of aqueous CO_2_ and bicarbonate ion. Thus, elevated rates of calcification associated with constant pH, inferred from coccolith *δ*^11^B, and elevated DIC, due to increased *p*CO_2_, at the site of calcification could also drive elevated rates of photosynthesis and result in constant PIC/POC ratios, i.e., constant rates of calcification relative to organic matter production, across *p*CO_2_ treatments.

### Stable carbon and oxygen isotopes within *O. neapolitana*

The *δ*^13^C_PIC_ and *δ*^13^C_POC_ range from −20‰ to 5‰ and from −45‰ to −20‰, respectively. The introduction of different mixtures of compressed air and isotopically light compressed CO_2_ to the experimental treatments resulted in seawater DIC *δ*^13^C (*δ*^13^C_DIC_) being relatively depleted in the 226 and 521 µatm treatment. Therefore, stable carbon isotope analyses were normalized to *δ*^13^C_DIC_, such that *δ*^13^C_PIC_ − *δ*^13^C_DIC_ = ∆^13^C_PIC_ and *δ*^13^C_POC_ − *δ*^13^C_DIC_ = ∆^13^C_POC_ (see Methods for details).

**Fig. 3:**
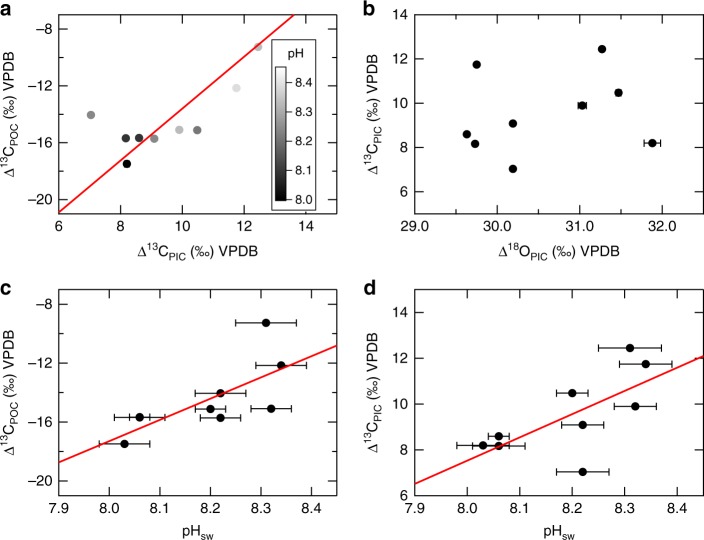
Stable isotope of carbon and oxygen in *O. neapolitana*. Comparisons between the carbon isotopic compositions of particulate organic and inorganic carbon (**a**), the carbon and oxygen isotopic compositions of particulate inorganic carbon (**b**), seawater pH and the carbon isotopic composition of particulate organic carbon (**c**), and seawater pH and the carbon isotopic composition of particulate inorganic carbon (**d**). Red lines represent linear regression lines of each panel. Error bars are 1 SD

The normalized ∆^13^C_PIC_ and ∆^13^C_POC_ range from 7‰ to 13‰ and from −18‰ to −9‰, respectively, with ∆^13^C_PIC_ consistently heavier than ∆^13^C_POC_ by about 24‰, and with ∆^13^C_PIC_ and ∆^13^C_POC_ exhibiting positive correlation (*R*^2^ = 0.56, *p* = 0.02; Fig. 3a). Both ∆^13^C_PIC_ (*R*^2^ = 0.47, *p* = 0.04) and ∆^13^C_POC_ (*R*^2^= 0.51, *p* = 0.03) are also positively correlated with seawater pH (Fig. 3c, d). Coccolith *δ*^18^O (*δ*^18^O_PIC_) range from −3.5‰ to −0.5‰. After normalizing to culture solution *δ*^18^O (*δ*^18^O_sw_), ∆^18^O_PIC_ (∆^18^O_PIC_ = *δ*^18^O_PIC_ − *δ*^18^O_sw_; VPDB (Vienna Pee Dee Belemnite)) ranges from 29.7 to 31.9‰, with no statistically significant correlation between ∆^18^O_PIC_ and ∆^13^C_PIC_, Fig. 3b.

**Fig. 4:**
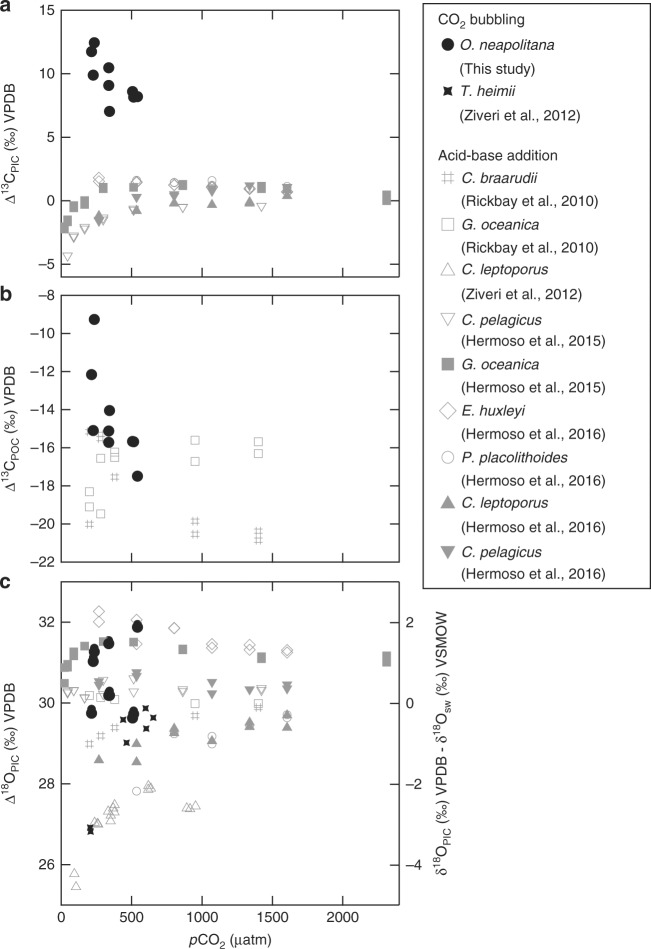
Stable carbon and oxygen isotopes of *O. neapolitana* compared to other coccolithophore species. Atmospheric *p*CO_2_ vs. Δ^13^C_PIC_ (**a**), Δ^13^C_POC_ (**b**), and Δ^18^O_PIC_ (**c**) for *O. neapolitana* (black solid circles) and other coccolithophore species. Δ^18^O_PIC_ and most Δ^13^C_POC_ data for *O. neapolitana* fall within the range reported for other species, with Δ^13^C_PIC_ being markedly higher. Offsets may be attributable to differences in species and/or method of acidification, as most past studies manipulate seawater pH via acid–base addition (labeled in gray), while the present study varied seawater pH via CO_2_ gas manipulation. Both the trends and absolute values of stable carbon and oxygen isotopes of *O. neapolitana* are compatible with the framework recently proposed by McClelland et al.^52^, as expanded on in Fig. 5 and the main text

Stable isotope results for both seawater and coccoliths were normalized to the VPDB scale, therefore, the reported ∆^18^O_PIC_ values are about 30‰ higher than previously published analyses of coccolithophores normalized to SMOW (e.g., refs.^10,46–49^) (Fig. 4). Values of ∆^13^C_POC_ and ∆^13^C_PIC_ in the present study are also slightly higher than previously reported values for cultured coccolithophores, particularly for ∆^13^C_PIC_, which is about 5–10‰ higher than previously published values^10,48–50^. This observed offset could be explained by differences in the experimental setup and/or species-and/or strain-specific carbon isotope fractionation.

Earlier work by Rickaby et al.^10^ and Hermoso et al.^49^ used HCl and NaHCO_3_ addition to manipulate seawater carbonate chemistry, while maintaining constant seawater pH in all treatments. Rost et al.^51^ and Hermoso^48^ used the acid–base addition method to recreate various seawater pH conditions, but with constant DIC. A ca. 3‰ variation in carbon isotope composition of coccolith calcite was observed for *E. huxleyi* cultured under similar *p*CO_2_ conditions, which Hermoso et al.^49^ attributed to different carbonate system parameters of the culture solutions arising from different modes of acidification, i.e., acid–base addition vs. CO_2_-bubbling, and other differences in experimental configuration. However, these factors should only account for 30–50% of the offset relative to prior studies in carbon isotopic compositions observed in the present study, suggesting that other factors are involved.

**Fig. 5:**
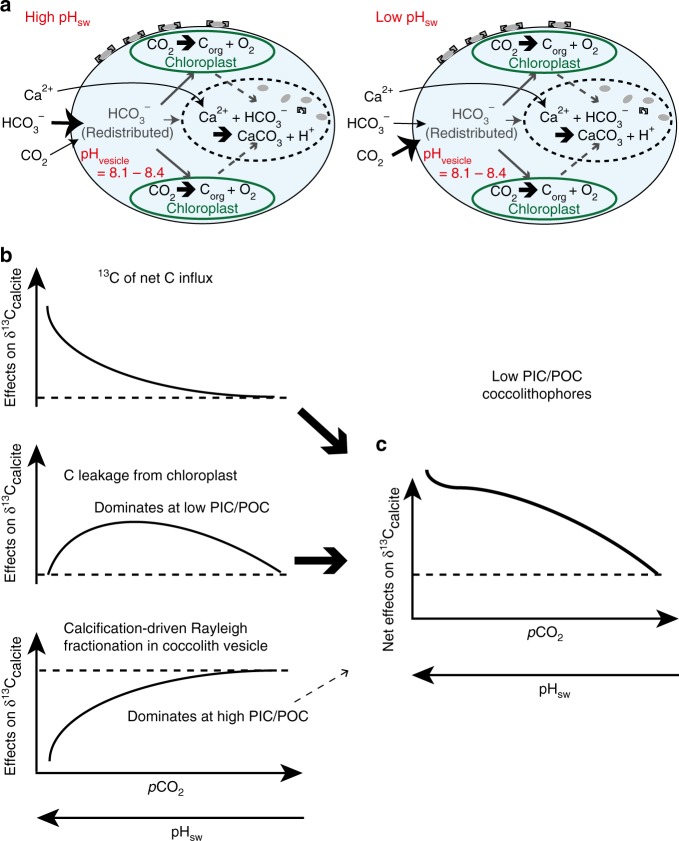
Schematic illustrations of inorganic carbon uptake by *O. neapolitana*. **a** Illustration of inorganic carbon uptake model proposed in this study for *O. neapolitana* under high and low pH_sw_ scenarios, which result in different proportions of CO_2_ to HCO_3_^−^ entering the cell. We hypothesize that the changing *δ*^13^C of inorganic carbon influx (driven by a higher proportion of CO_2_ to HCO_3_^−^ entering the cell under lower pH_sw_) dominates the carbon isotope fractionation recorded in *O. neapolitana*. In both scenarios, coccolith vesicle pH is constant despite varying seawater pH, as suggested by the relatively fixed boron isotope composition of the coccoliths formed over a range of seawater pH. Notably, pH homeostasis in the calcifying vesicle and changing carbon supply are consistent with the lack of differences in growth rates across seawater treatments, as suggested by the lack of observable changes in PIC/POC ratio and size of coccoliths and coccospheres. **b** The individual effects of changing inorganic carbon influx, carbon leakage from chloroplast, and calcification-driven Rayleigh fractionation of the calcifying fluid on *δ*^13^C_PIC_ (modified from McClelland et al.^52^). **c** The combined effects of changing inorganic carbon influx, carbon leakage from chloroplast, and calcification-driven Rayleigh fractionation of the calcifying vesicle fluid on *δ*^13^C_PIC_ in low PIC/POC coccolithophore species. Note that the calcification-driven Rayleigh fractionation has a relatively small effect for low PIC/POC coccolithophores, as indicated by arrow size

Species-specific differences in carbon isotope fractionation might also contribute to the differences observed between the present and past studies^10,48,49^. McClelland et al.^52^ proposed a model for evaluating potential sources of inorganic carbon for coccolithophores, as illustrated in Fig. 5b. The model predicts that coccolithophore species with lower PIC/POC ratios, such as *P. placolithoides*, will have a higher stable carbon isotopic composition in coccolith calcite under lower aqueous CO_2_ conditions due to increased flux of isotopically heavy carbon leaking from the chloroplast that is utilized in calcification and photosynthesis (Fig. 5c). This effect is predicted to decrease at higher-CO_2_ levels, which is consistent with the convergence between *O. neapolitana* *δ*^13^C and *δ*^13^C of other species cultured under higher-CO_2_ conditions. Although there are currently no published carbon isotope data for coccolithophores cultured at *p*CO_2_ <500 µatm, the McClelland model predicts a ∆^13^C_PIC_ for *P. placolithoides* as high as 3‰ under [CO_2(aq)_] of ~5 µM. In summary, coccolithophores with different PIC/POC ratios should show species-specific carbon isotopic fractionation within the same [CO_2(aq)_] range. Therefore, this model at least partially explains the larger carbon isotope fractionation observed in the present study.

Differences in acidification methods used in published experiments, i.e., acid–base addition vs. CO_2_-bubbling, which results in differential DIC speciation may also explain the differing carbon isotope fractionation by coccolithophores shown in this study compared to prior studies. The present study acidified seawater in experimental treatments by sparging treatments with mixed gases formulated at different *p*CO_2_, which yielded a fixed TA, elevated DIC, and reduced pH. In contrast, acidifying the seawater treatments via acid addition decreases TA, DIC, and pH. Thus, the two approaches yield different concentrations of total DIC and DIC speciation for equivalent seawater pH treatments. For the lowest pH treatments, the CO_2_-gas sparging method for acidification yields up to 50% higher concentrations of DIC than the acid-addition method of acidification. For the highest pH treatments, the CO_2_-gas sparging method of acidification maintains lower concentrations of HCO_3_^−^ than the acid-base addition method. Therefore, the differential speciation of DIC between equivalent experimental treatments acidified using different methods combined with differences in the stable carbon isotopic composition of different DIC species could yield differences in coccolithophore ∆^13^C between the different experiments. Although this is unlikely to fully explain the 5–10‰ higher stable carbon isotopic compositions in both organic and inorganic portions of *O. neapolitana*, the combination of these effects with species-level differences in carbon isotope fractionation may account for the observed discrepancies in ∆^13^C between the present and prior studies (e.g., refs.^51,53^).

The positive correlation between ∆^13^C_PIC_ and ∆^13^C_POC_ (Fig. 3a) suggests that the carbon sources for calcification and photosynthesis are primarily derived from the same internal DIC pool, and/or that the carbon sources for both processes exhibit the same response to changing seawater pH. Both ∆^13^C_PIC_ and ∆^13^C_POC_ become about 6–10‰ lighter with the prescribed decrease in seawater pH and increase in seawater DIC. Hermoso et al.^49^ suggest that under elevated DIC, photosynthesis-driven Rayleigh fractionation of carbon isotopes may be diluted by the larger carbon pool, resulting in lower *δ*^13^C for *E. huxleyi* calcite. However, a decrease in *δ*^13^C with decreasing seawater pH was also observed for the organic portions of coccolithophores in the present study, and this cannot be explained by an increased DIC pool. It is therefore likely that other mechanisms control stable carbon isotopic fractionation within this species, at least for the organic portion of the algae.

Recent studies suggest that *E. huxleyi* has different CO_2_ and HCO_3_^−^ influxes under different seawater pH conditions^54,55^. Although a different species was investigated in the present study, one explanation for the observed correlation between seawater pH and both ∆^13^C_PIC_ and ∆^13^C_POC_ is that the carbon sources for calcification and photosynthesis changed under acidified conditions, potentially because more CO_2_ was transported across the cell membrane compared with HCO_3_^−^. When pH decreases from 8.5 to 7.9, *δ*^13^C of different DIC species will be 1‰ lighter due to the re-partitioning of species in the DIC system^56^. Additionally, assuming equilibrium isotope fractionation, *δ*^13^C of CO_2(aq)_ is about 9‰ lower than that of HCO_3_^−^ for the same *δ*^13^C_DIC_ at 25 °C and 35 PSU^56^. The CO_2_ gradient across the coccolithophore cell membrane increases as *p*CO_2_ increases. Therefore, when a greater portion of CO_2_, compared with HCO_3_^−^, is transported across the cell membrane, the *δ*^13^C of the coccolithophore internal DIC pool will decrease. Thus, the observed correlation between seawater pH and both ∆^13^C_PIC_ and ∆^13^C_POC_ is consistent with the hypothesis that substantially more CO_2_ is taken into the internal DIC pool of *O. neapolitana*, which supplies carbon for both its calcification and photosynthesis, at lower seawater pH.

Instead of (or in addition to) increasing CO_2_ influx, the observed depletion in ^13^C with decreasing seawater pH can also be explained by reduced uptake of HCO_3_^−^ in a higher DIC environment. In either case, more ^13^C-depleted inorganic carbon is incorporated into the internal DIC pool of *O. neapolitana* for calcification and photosynthesis, resulting in positive correlations between ∆^13^C_PIC_ and pH_sw_, between ∆^13^C_POC_ and pH_sw_, and therefore between ∆^13^C_PIC_ and ∆^13^C_POC_. These carbon species are then redistributed in the algae’s ^13^C-depleted internal medium and utilized for both calcification and photosynthesis. Assuming the two processes use DIC from the same internal pool, the inorganic and organic carbon isotopic compositions would fractionate relative to the same *δ*^13^C_DIC_. However, a greater difference in ∆^13^C_POC_ (ca. 8‰) was observed between the two end-member *p*CO_2_ treatments (226 and 521 µatm) compared to the end-member differences in ∆^13^C_PIC_ (6‰), suggesting that additional fractionation occurs within chloroplasts and/or calcification vesicles.

McClelland et al.^52^ hypothesized in a recent study that the *δ*^13^C composition of coccolith calcite is influenced by the *δ*^13^C composition of carbon entering the cell, leakage of carbon from chloroplasts, and Rayleigh-type fractionation of carbon isotopes in the calcifying vesicle. They proposed that the *δ*^13^C compositions of the carbon influx and carbon leakage from chloroplasts have stronger effects in coccolithophore species with lower PIC/POC ratios. One explanation for the different magnitudes of pH-induced offsets in ∆^13^C between the organic and inorganic portions of coccolithophores is that isotopically heavier carbon leaked from the chloroplast may partially compensate for the decline in *δ*^13^C resulting from the increasing CO_2_ influx. Otherwise, the 1:1 slope of the regression between ∆^13^C_POC_ and ∆^13^C_PIC_ (slope = 1.01) indicates that the pattern resulted from the change in *δ*^13^C composition of a single DIC pool that supplies carbon for both calcification and photosynthesis within the algae.

A carbonate ion effect on CaCO_3_ *δ*^18^O has been both hypothesized and observed in biominerals, such that CaCO_3_ *δ*^18^O will decrease with increasing pH because of the resulting increase in concentration of CO_3_^2−^, which has a more depleted *δ*^18^O composition^57^. This effect has been observed in foraminifera, corals^58^, and coccolithophores^47^. However, the opposite trend, albeit weak (*p* > 0.05), was observed between *δ*^18^O of *O. neapolitana* coccolith calcite and culture seawater pH in the present study, with coccolith *δ*^18^O increasing by ca. 2‰ as pH increased from 8.0 to 8.3. This may indicate that although inorganic carbon uptake changes under the different *p*CO_2_ treatments, the relative abundance of DIC species at the site of calcification is not significantly different due to active biological control of carbonate chemistry within the calcifying vesicle. Alternatively, the conditions of calcification within this species may not preserve isotopic fractionation that arises from DIC speciation in its calcifying fluid if, for example, mineral precipitation is too slow^59^ or if there is additional unconstrained biological fractionation affecting oxygen isotope compositions.

### No PIC/POC changes in response to elevated *p*CO_2_

**Fig. 6:**
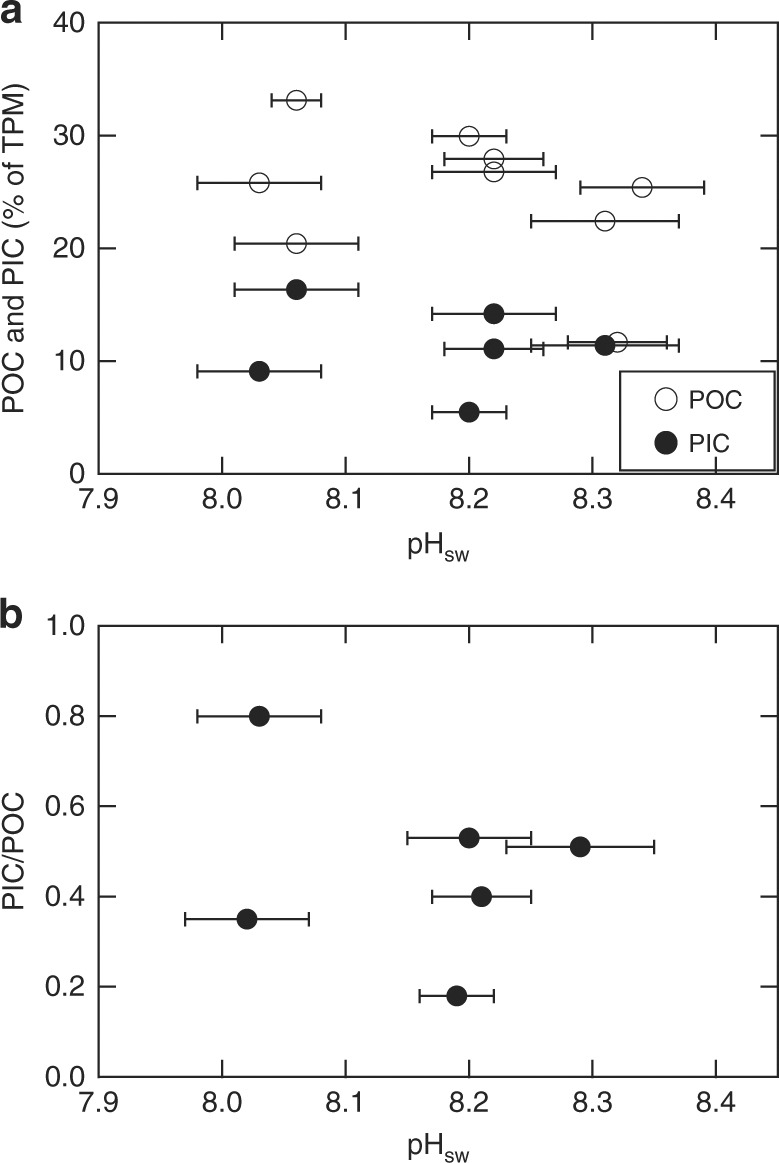
PIC, POC, and PIC/POC of *O. neapolitana* for varying pH_SW_. Seawater pH vs. particulate inorganic carbon (PIC) content as weight percent of total particulate matter (TPM) (solid circle, **a**), particulate organic carbon (POC) content as weight percent of TPM (open circle, **a**), and PIC/POC ratio (**b**). Error bars are 1 SD

The particulate organic carbon (POC) and particulate inorganic carbon (PIC) content range from 11% to 34% and from 5% to 17%, respectively, of the coccolithophore total particulate matter (TPM) weight (Fig. 6a). The PIC/POC ratios range from 0.18 to 0.80 (mean ± 1*σ* = 0.46 ± 0.21; *n* = 6). No statistically significant trend was observed between pH and either PIC (*p* = 0.80) or POC (*p* = 0.29) content, or the PIC/POC ratio (*p* = 0.70) (Fig. 6b).

The lack of correlation between ambient pH_sw_ and PIC, POC, or PIC/POC suggests that *O. neapolitana* does not change the proportions of DIC allocated for calcification and photosynthesis with increasing *p*CO_2_. Although there are no previously published PIC/POC ratios for *O. neapolitana*, this result has been observed in the coccolithophore species *Pleurochrysis carterae*, which also precipitates high-Mg calcite^6,34^. Casareto et al.^6^ observed increases in both PIC and POC in cultures at 1200 µatm *p*CO_2_ and at ambient *p*CO_2_ after 7 days of growth. However, higher production of POC than PIC yields PIC/POC ratios that are lower than the PIC/POC ratio in the beginning of the experiment for both *p*CO_2_ treatments, but no significant difference between the two *p*CO_2_ treatments. The similar PIC/POC trends when pH deceases in the two high Mg/Ca coccolith species suggest that coccolithophores with high Mg/Ca ratios may have different mechanisms for controlling the allocation of inorganic carbon for photosynthesis and calcification from other low-Mg coccolithophore species. For instance, it was previously hypothesized that coccolithophore species producing high-Mg calcite (mMg/Ca >0.4) in modern seawater with elevated Mg/Ca ratio (mMg/Ca = 5.2), which favors the abiotic precipitation of high-Mg calcite over low-Mg calcite (mMg/Ca <0.4), have less control over the ionic composition of their calcifying medium than coccolithophores that produce low-Mg calcite in seawater favoring the precipitation of high-Mg calcite^60^.

Strontium isotope ratios of coccoliths (Supplementary Note 2 and Supplementary Fig. 1) also show no variation across pH treatments. Stevenson et al.^61^ identified a correlation between coccolithophore growth rate (co-varying with temperature) and coccolith *δ*^88/86^Sr for the species *E. huxleyi*, *Coccolithus pelagicus* spp. *braarudii*, and *Gephyrocapsa oceanica*. The lack of a statistically significant difference in *δ*^88/86^Sr between pH treatments in the present experiment on *O. neapolitana* is consistent with the observation that PIC/POC ratios are invariant across pH treatments.

### Scanning electron microscopy images of coccoliths

**Fig. 7:**
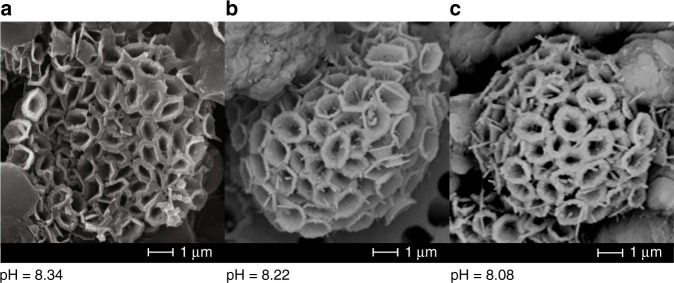
Scanning electron images of typical *O. neapolitana* coccolithophores from the three *p*CO_2_ treatments. Electron images do not reveal differences in sizes of coccoliths and coccospheres under different *p*CO_2_ treatments. The effective culture seawater pH values are (**a**) 8.34, (**b**) 8.22, and (**c**) 8.08. Scale bars are 1 μm in all images

Imaging of coccoliths via scanning electron microscopy (SEM) does not reveal any gross changes in diameter or thickness of the individual coccoliths or of the diameter of the coccolithophore sphere as a whole (Fig. 7). Coccolithophore spheres are about 6–8 µm in all treatments, which is consistent with size ranges previously reported for these species^29^. The consistent size of coccoliths observed via SEM, the constant PIC/POC ratios, and the boron isotope results support the assertion that calcification, relative to organic matter production, within *O. neapolitana* is unimpeded by elevated *p*CO_2_ due to maintenance of pH homeostasis at the alga’s site of calcification.

### Mechanisms for *O. neapolitana* adaptation to increasing *p*CO_2_

The results of the present study suggest that calcification and photosynthesis within the coccolithophore *O. neapolitana* utilize carbon from the same internal DIC pool (Fig. 5a). The preferential uptake of light isotopes by the rubisco enzyme during photosynthesis results in ca. 25‰ offset between *δ*^13^C_PIC_ and *δ*^13^C_POC_ in this species. When seawater DIC increases as a result of increasing atmospheric-*p*CO_2_, ^13^C becomes depleted in the internal DIC pool either through increased uptake of dissolved CO_2_ and/or decreased uptake of HCO_3_^−^, thereby decreasing both *δ*^13^C_PIC_ and *δ*^13^C_POC_. The observation that coccolith *δ*^11^B remains constant across a range of seawater pH suggests that *O. neapolitana* maintains a fixed pH at the site of calcification across this range of seawater pH. This enables the coccolithophores to continue producing coccoliths under lower pH and higher DIC conditions. Additionally, PIC, POC, and PIC/POC ratio were statistically invariant under all *p*CO_2_ conditions, indicating that *O. neapolitana* maintained similar rates of calcification, relative to rates of photosynthesis, irrespective of *p*CO_2_ treatment, even though the energetic cost of calcification is likely increasing with declining seawater pH. Furthermore, SEM imaging reveals that coccoliths formed under the high-*p*CO_2_ treatments were similar in size and shape to coccoliths formed in the control and low-*p*CO_2_ treatments. These results are consistent with prior studies showing that some species of coccolithophores are able to maintain relatively constant ratios of calcification-to-photosynthesis under conditions of elevated *p*CO_2_^5,9,62^. The present study shows that some species of coccolithophores achieve this by maintaining pH homeostasis at the site of calcification and by utilizing a greater proportion of the isotopically lighter CO_2_ species of DIC for both photosynthesis and calcification.

## Methods

### Culture conditions and seawater carbonate chemistry

The coccolithophore *O. neapolitana* was cultured in a synthetic seawater medium made from a commercial sea salt mixture (Instant Ocean Sea Salt^63^) enriched with nutrients following the F/2 recipe^64^ from 11 January 2012 to 30 January 2012 at the University of North Carolina, Chapel Hill. Algal cultures were divided between three *p*CO_2_ treatments in triplicate aquaria, equilibrated with air-CO_2_ mixtures of 280, 400, and 750 p.p.m., selected to mimic levels before the industrial revolution, current atmospheric conditions, and levels predicted for year 2100 AD, resulting in seawater pH (±SE) of 8.05 ± 0.01, 8.22 ± 0.01, and 8.33 ± 0.01 and effective *p*CO_2_ (±SE), calculated from measured DIC and TA, of 226 ± 11, 339 ± 10, and 521 ± 19. The nominal 280 µatm *p*CO_2_ gas was formulated by mixing compressed CO_2_ gas and compressed CO_2_-free air, the nominal 400 µatm *p*CO_2_ gas was compressed ambient air, and the nominal 750 µatm *p*CO_2_ gas was formulated by mixing compressed CO_2_ gas and compressed ambient air. Gases were mixed with Aalborg solenoid-valve-based mass flow controllers. The cultures were maintained at constant temperature (25 °C) and salinity (35 PSU) on a 16–8 h light–dark cycle in 38 l glass aquaria with the same initial dose of coccolithophore cells (~400 ± 40 cell per l). The level and duration of the diurnal lighting cycle was based on Langer et al.^9^, Rickaby et al.^10^, Riebesell et al.^21^, Langer et al.^62^, Krug et al.^65^, and Zondervan et al.^66^ Temperature and salinity were based on the natural conditions of the source cultures. Nutrients in the experimental cultures were formulated pursuant to the f/2-si media, based upon Langer et al.^9^, Langer et al.^62^, Guillard and Ryther^64^, Krug et al.^65^, Zondervan et al.^66^, and Guillard^67^. Cultures were extremely dilute and collected at cell concentrations <100,000 cell per ml, which places culture growth well below the stationary phase^34^.

Temperature, salinity, and pH were measured every other day in the experimental tanks. For temperature measurements, a NIST (National Institute of Standards and Technology)-calibrated partial-immersion organic-filled glass thermometer (precision ± 0.3%, accuracy ± 0.4%) was used. A YSI 3200 conductivity meter with a YSI 3440 cell (*K* = 10) calibrated with seawater standards (A. Dickson, Scripps Institute of Oceanography) was used for salinity. Seawater pH was measured with an Orion 9156BNWP pH probe. The instrument was calibrated with two Orion NBS buffers (7.00 and 10.01) traceable to NIST standard reference material (for slope of the calibration curve) and with seawater standards of known pH (A. Dickson, Scripps Institute of Oceanography; for *y*-intercept of the calibration curve). Seawater DIC and TA were measured every week via coulometry (UIC 5400) and via closed-cell potentiometric Gran titration calibrated with certified Dickson TA/DIC standards, respectively (see Supplementary Table 1 for seawater chemistry data). The certified reference materials (CRMs) were monitored during the measurements and the experimental seawater DIC and TA measurements were all corrected to the certified values of CRMs.

To determine seawater *p*CO_2_, pH, carbonate ion concentration, [CO_3_^2−^], bicarbonate ion concentration, [HCO_3_^−^], and aqueous CO_2_, the program CO2SYS^68^ was used with the inputs of weekly DIC and TA measurements and weekly average of temperature and salinity. The K_1_ and K_2_ carbonic acid constants from Roy et al.^69^, the stoichiometric aragonite solubility product from Mucci^70^, and an atmospheric pressure of 1.015 atm were applied in the program for the full seawater chemistry calculations (Supplementary Table 1).

Although a short-term rise in pH was observed in all treatments during the last week of culturing (see Supplementary Note 3 and Supplementary Fig. 2 for carbonate chemistry of the culture media), all isotopic and PIC/POC data are compared to the experiment-long average of the calculated values of seawater pH (pH_sw_), which were stable throughout most of the experiment.

Coccolithophores were collected after 2 weeks of culturing, which yielded 10–15 generations of coccolithophores and ensured acclimation to the treatment conditions. The coccolithophores were filtered from the seawater, rinsed with 95% ethanol (95% ethanol and 5% deionized water), and stored in 95% ethanol to prevent organic decay or dissolution. Additional culturing methods are provided in the supplementary materials.

### Chemical pretreatments

Samples were cleaned and prepared for chemical analysis under class 10 laminar flow hoods in a class 10,000 clean room in the Glaciochemistry and Isotope Geochemistry Laboratory at the University of Michigan’s Department of Earth and Environmental Sciences. About 10 µl of the coccolith-ethanol mixture was set aside for SEM examination. Cleaning protocols and chemical pretreatments for isotopic analysis followed the methods of Barker et al.^71^ and Liu et al.^72^ Coccolithophore samples were rinsed three times with Super-Q (SQ) water (Millipore, >18.2 MΩ•cm) and then split three ways: 1.0–1.5 mg of dry sample was allocated for total carbon (TC); 0.5–1.0 mg of dry sample was allocated for total organic carbon; and at least 2 mg of dry sample was allocated for carbon, oxygen, boron, and strontium stable isotope analysis. Samples for TC and total organic carbon analysis were treated overnight with 2% HCl to remove calcium carbonate and then were rinsed three times with SQ water. Samples for inorganic elemental and isotopic analysis were treated overnight with 10% H_2_O_2_ buffered with NaOH. After centrifugation and decanting of the solution, samples were rinsed with SQ water, 0.001 N HNO_3_, and SQ water again to prevent recoil of organics or dust onto the samples. About 30–100 µg of clean sample was prepared for inorganic *δ*^13^C and *δ*^18^O analyses. At least 1 mg of cleaned sample was dissolved into 1.7 N HCl for a target [B] of ca. 750 p.p.b.

### Analysis of carbon and oxygen isotopes

TC was analyzed using a Costech ECS 4010 elemental analyzer in the Oceanography and Marine Geology Laboratory at the University of Michigan’s Department of Earth and Environmental Sciences. Samples were weighed and wrapped in tin foil capsules for combustion. An acetanilide standard was used to calibrate carbon detection with respect to sample weight. Reproducibility in weight percent was 70.83 ± 1.08% (1*σ*, *n* = 6).

Total organic carbon content and *δ*^13^C were analyzed at University of Michigan’s Stable Isotope Laboratory on a Costech ECS 4010 elemental analyzer coupled to the inlet of a Finnigan Delta V Plus mass spectrometer operating in continuous flow mode. About 200–600 µg of organic coccolithophore sample was weighed in tin foil capsules. An acetanilide standard was used to calibrate organic carbon weight percent, and additional IAEA 600 caffeine and IAEA-CJ-6 sucrose standards were used to calibrate the organic *δ*^13^C analyses. The acetanilide standard measured 71.26% with a reproducibility of 0.95% (*n* = 11). The organic *δ*^13^C for the acetanilide, IAEA 600 caffeine and IAEA-CJ-6 sucrose standards were measured as −33.75 ± 0.07‰, −27.77 ± 0.06‰, and −10.45 ± 0.05‰, respectively.

The *δ*^13^C and *δ*^18^O of coccolith calcite were analyzed with a Finnigan MAT Kiel IV carbonate preparation device coupled directly to the inlet of a Finnigan MAT 253 triple collector isotope ratio mass spectrometer in the Stable Isotope Laboratory at University of Michigan’s Department of Earth and Environmental Sciences. The precisions of measurements were monitored with NBS 19 limestone standard (*δ*^13^C = 1.96 ± 0.07‰ and *δ*^18^O = −2.24 ± 0.07‰; 1*σ*, *n* = 10). The *δ*^18^O of culture seawater was analyzed with a Picarro L2120-i Cavity Ringdown Spectrometer equipped with the A0211 high-precision vaporizer, autosampler, and ChemCorrect software at the University of Michigan Water Isotopes Lab. No water samples were preserved onsite for stable carbon analysis. The *δ*^13^C_DIC_ values used for ∆^13^C_POC_ and ∆^13^C_PIC_ were based on measurements of seawater solutions that were formulated similarly to those used in the culture experiment. The *δ*^13^C_DIC_ of seawater bubbled with compressed ambient air was −5.90‰ (450 µatm at 30 °C) and −6.67‰ (540 µatm at 20 °C). The *δ*^13^C_DIC_ of seawater bubbled with mixtures of compressed ambient air and compressed CO_2_ was −13.83‰ (750 µatm at 30 °C) and −15.69‰ (830 µatm at 20 °C). The *δ*^13^C_DIC_ of the compressed CO_2_ gas was calculated from the above results using mass balance equations, and resulted in an average *δ*^13^C_DIC_ of −29.09‰ for the compressed CO_2_ gas. The average *δ*^13^C_DIC_ of the 20 and 30 °C seawaters bubbled with compressed ambient air (−6.29‰) and the average *δ*^13^C_DIC_ of compressed CO_2_ gas (−29.09‰) were then calibrated to 25 °C and the pH of each treatment following the method described in Zeebe and Wolf-Gladrow^56^. The seawater *δ*^13^C_DIC_ values used in this study were calculated as −28.98‰, −6.29‰, and −14.42‰ for water treated with mixture of compressed CO_2_ and compressed CO_2_-free air (low-*p*CO_2_ condition), compressed ambient air (present-day *p*CO_2_ condition), and mixture of compressed CO_2_ and compressed ambient air (high-*p*CO_2_ condition). Although this approach to estimating seawater *δ*^13^C_DIC_ may yield results that deviate slightly from direct measurements of seawater *δ*^13^C_DIC_, it should not materially alter the trends in the carbon isotopic data, nor their interpretation. The seawater *δ*^18^O values were calibrated to VSMOW/VSLAP using three internal laboratory standards, with analytical precision (1*σ*) better than 0.1‰. To calculate ∆^18^O_PIC_ (∆^18^O_PIC_ = *δ*^18^O_PIC_ − *δ*^18^O_sw_), seawater *δ*^18^O was converted to VPDB scale with the relationship: *δ*^18^O_VPDB_ = 0.97001 × *δ*^18^O_VSMOW_ − 29.99 (‰)^73,74^.

All coccolith *δ*^13^C and *δ*^18^O results are reported relative to the VPDB standard:

1 $${\mathrm{\delta }}^{13}{\mathrm{C}} = \left[ {\frac{{\left( {\,^{13}{\mathrm{C}}{/}^{12}{\mathrm{C}}} \right)_{{\mathrm{sample}}}}}{{\left( {\,^{13}{\mathrm{C}}{/}^{12}{\mathrm{C}}} \right)_{{\mathrm{VPDB}}}}} - 1} \right] \times 1000\left( {{ \textperthousand }} \right)$$

2 $${\mathrm{\delta }}^{18}{\mathrm{O}} = \left[ {\frac{{\left( {\,^{18}{\mathrm{O}}{/}^{16}{\mathrm{O}}} \right)_{{\mathrm{sample}}}}}{{\left( {\,^{18}{\mathrm{O}}{/}^{16}{\mathrm{O}}} \right)_{{\mathrm{VPDB}}}}} - 1} \right] \times 1000({{ \textperthousand }})$$

### PIC/POC

Measurements and calculations of PIC and POC were made using standard procedures (e.g., refs.^75,76^). Coccolithophore samples from each treatment were dehydrated and weighed to determine their TPM weight. The particulate inorganic matter (PIM), i.e., CaCO_3_, in each sample was dissolved by addition of 2% HCl. Each sample was then re-weighed after removing the PIM, yielding the particulate organic matter (POM) weight. TC (%) of the non-acidified samples and POC (%) of the acidified samples were measured with a Costech Elemental Analyzer attached to an IRMS (the latter was used for carbon isotope measurements described in the carbon isotope methods section). Carbon-loss measurements were verified with acetanilide (*C* = 71.26 wt%) and atropine standards (*C* = 70.83 wt%). Each TC weight was calculated by multiplying TC (wt%) by the TPM weight, and POC weight was calculated by multiplying the (wt%) carbon in POM by the POM weight. PIC weight was then calculated as the difference between TC weight and POC weight. POC (wt%) and PIC (wt%) were then calculated by, respectively, dividing the POC and PIC weights by the TPM weight. The PIC/POC ratio was calculated by dividing PIC (wt%) by POC (wt%). Three of the calculated PICs (wt%) were eliminated due to weak signal-to-noise ratios of their TC (wt%) measurements.

### Boron isotopes

The boron isotopic composition of both culture solution and coccolith samples were measured following the method of Liu et al.^72^ Briefly, samples were dissolved in 1.7 N HCl to achieve a [B] = ca. 750 p.p.b. The microsublimation technique was used to separate boron from its matrix, which exploits boron’s high volatility compared to other elements and organic compounds. About 50 μl of dissolved sample solution was loaded onto the cap of a conical bottom Savillex^®^ 5 ml vial. The vial was then closed and placed up-side down on a 65–70 °C patented extraction plate^77^ for 12 h. Boron isotopic analyses were conducted using a Thermo Fisher Triton PLUS multicollector thermal ionization mass spectrometer operating in negative ion mode for boron isotope analysis in the Glaciochemistry and Isotope Geochemistry Lab (GIGL) at the University of Michigan’s Department of Earth and Environmental Sciences. Purified sample solution, 1 μl, was loaded onto an outgassed refilament after loading 1 μl of boron-free seawater. Boron analyses were conducted using the total evaporation method^78^. Boron isotopic composition in seawater and coccolith samples were reported as *δ*^11^B, where

3 $${\mathrm{\delta }}^{11}B = \left[ {\frac{{\left( {\,^{11}{\mathrm{B}}/^{10}{\mathrm{B}}} \right)_{{\mathrm{sample}}}}}{{\left( {\,^{11}{\mathrm{B}}/^{10}{\mathrm{B}}} \right)_{{\mathrm{SRM}}\;951{\mathrm{a}}}}} - 1} \right] \times 1000( \textperthousand )$$

with ^11^B/^10^B for the boric acid standard SRM 951a is 4.0345 ± 0.0084‰ (2*σ*, *n* = 24). The reproducibility (2*σ*) of *δ*^11^B in seawater and carbonate samples were 1.13 and 1.83‰, respectively^79^. A *δ*^11^B-pH transfer equation was used to calculate coccolith vesicle pH:

4 $${\mathrm{pH}} = {{pK}}_{\mathrm{b}} - {\mathrm{log}}\left( {\frac{{{\mathrm{\delta }}^{11}{\mathrm{B}}_{{\mathrm{sw}}} - {\mathrm{\delta }}^{11}{\mathrm{B}}_{{\mathrm{carbonate}}}}}{{\alpha {\mathrm{\delta }}^{11}{\mathrm{B}}_{{\mathrm{carbonate}}} - {\mathrm{\delta }}^{11}{\mathrm{B}}_{{\mathrm{sw}}} + 1000\left( {\alpha - 1} \right)}}} \right),$$

where *pK*_b_ and *α* are the boron equilibrium constant and boron fractionation factor, respectively. The *pK*_b_ of each sample was calculated from average temperature and salinity of each replicate tank following the equation described by the US Department of Energy^80^, using an *α* of 1.0272^43^. Temperature, salinity, and total seawater boron *δ*^11^B of each replicate tank were used to calculate calcifying vesicle pH in the different treatments.

The reproducibility of *δ*^11^B in carbonate samples is about 1.8‰, which can be converted to about 0.18 pH unit. Although this means that the *δ*^11^B analyses cannot resolve pH variations within 0.18 units, it is sufficient to detect the potential change in coccolith *δ*^11^B if coccolith *δ*^11^B was simply responding to the prescribed 0.3 unit difference in seawater pH across the experimental treatments.

### Scanning electron microscopy

SEM images of coccolithophores from different treatments were obtained with a Hitachi S3200N Scanning Electron Microscope at the University of Michigan’s Electron Microbeam Analysis Laboratory and a Phenom G2 pro Scanning Electron Microscope at Roscoff Marine Station. For images obtained at the University of Michigan, dry coccolith pellets were mounted with carbon tape and coated with gold for at least 120 s to prevent static charging of their surface. A 15 kV beam was applied to the samples with an 8 mm working distance. For images obtained at Roscoff Marine Station, samples were filtered on a membrane, which was then cut and mounted on the sample holder with carbon tape and imaged with a 10 kV beam. SEM images were obtained with a resolution better than 1024 × 800.

### Radiogenic and stable Sr isotopes

Analyses of radiogenic and stable Sr isotopic followed the procedure described in Liu et al.^79^ Briefly, the residuals of coccolith samples after boron microsublimation were re-dissolved in 7 N nitric acid. A ^87^Sr–^84^Sr double spike (spike:sample ratio = 1:1) was used to determine the stable strontium isotope composition of the culture medium and coccoliths. Sr was separated from other matrices by passing both unspiked and spiked samples through a 50–100 μm Sr-spec resin (Eichrom). About 100–200 ng and 200–250 ng of Sr in unspiked and spiked sample, respectively, were loaded on outgassed Re-single filament for Sr isotope analyses conducted on Thermo Fisher Triton PLUS multicollector thermal ionization mass spectrometer at the Glaciochemistry and Isotope Geochemistry Lab (GIGL) at the Department of Earth and Environmental Sciences, University of Michigan. To determine the Sr isotopic ratios and achieve within-run precision better than 10 p.p.m. (2 SE), a total of 400 cycles of data were collected for each measurement. The reproducibility of Sr isotopic standard SRM987 is 0.710246 ± 13 (2*σ*, *n* = 42). For direct comparisons to literature, all ^87^Sr/^86^Sr data reported in this study were normalized to SRM987 = 0.710250. The stable Sr data were reported as *δ*^88/86^Sr, defined as:

5 $${\mathrm{\delta }}^{88/86}{\mathrm{Sr}} = \left[ {\frac{{\left( {{1}^{88}{\mathrm{Sr}}{/}^{86}{\mathrm{Sr}}} \right)_{{\mathrm{sample}}}}}{{\left( {{1}{88}{\mathrm{Sr}}{/}^{86}{\mathrm{Sr}}} \right)_{{\mathrm{SRM}}\;987}}} - 1} \right] \times 1000({{11111}})$$

The reproducibility of *δ*^88/86^Sr values in the seawater standard IAPSO is 0.365 ± 73‰ (2*σ*, *n* = 4) and in the inter-laboratory carbonate standard JCp-1 is 0.195 ± 21‰ (2*σ*, *n* = 4).

### Data availability

All results of the culture experiment and isotopic analyses are presented in the manuscript and Supplementary Table 1. The data is also available upon request from the corresponding authors.

## Electronic supplementary material

Supplementary Information
