# Supplementary material for: A coastal coccolithophore maintains pH homeostasis and switches carbon sources in response to ocean acidification
Source: Nat Commun. 2018 Jul 20;9:2857. doi: 10.1038/s41467-018-04463-7 (PMC6054640; doi:10.1038/s41467-018-04463-7)
Supplement: Supplementary file 1 — Supplementary Information [file 41467_2018_4463_MOESM1_ESM.pdf]

Supplementary Information for "A coastal coccolithophore maintains pH homeostasis and switches carbon sources in response to ocean acidification" by Liu, Y.-W., *et al*.

Supplementary Note 1 Here we further consider permutation scenario 3 that different portions of boric acid and borate ion can transport into the cell vesicle and that they may change across experimental conditions. Each model result represents a fix proportion (P, value between 0 to 1) of seawater total boron entering the calcifying vesicle as boric acid. The boron isotopic composition in the cell vesicle can therefore be expressed as:

$$\delta^{11}\mathrm{B}_{\mathrm{vesicle}} = \left[\frac{\delta^{11}\mathrm{B}_{\mathrm{Boric\ acid}}\times[\mathrm{Boric\ acid}]_{\mathrm{sw}}\times P+\delta^{11}\mathrm{B}_{\mathrm{Borate}}\times[\mathrm{Borate}]_{\mathrm{sw}}\times(1-P)}{[\mathrm{Boric\ acid}]_{\mathrm{sw}}\times P+[\mathrm{Borate}]_{\mathrm{sw}}\times(1-P)}\right] \quad \text{(S1)}$$

, where $\delta^{11}B_{Boric\ acid}$, $[Boric\ acid]_{sw}$, $\delta^{11}B_{Borate}$, and $[Borate]_{sw}$ are boron isotopic composition of seawater boric acid, boric acid concentration in seawater, boron isotopic composition in seawater borate and borate concentration in seawater, respectively. The concentration and boron isotopic composition of seawater boric acid and borate transport into the cell vesicle therefore will change with respect to their nature distribution in seawater. That is, under low (high) pH, boric acid (borate) is dominant in seawater, and both of their boron isotopic composition increases as seawater pH increase. Therefore, if there is no discrepancy between seawater boric acid and borate transport in to coccolith vesicle, the proportion of boric acid and borate contribute to the boron isotopic composition in

coccolith vesicle is 0.50 and 0.50. To fit the data, the portion of boric acid attributed to the coccolith $\delta^{11}B$ value varies from 0.55 under pH = 8.35, to 0.50 under pH = 8.2, then to over 0.80 under pH = 8.05. We are not aware of evidence in any marine calcifying organisms to support the idea that the boric acid permeability or transport would change between 0.50 and 1.00 under different ambient pH conditions, or a proposed mechanism by which this would occur. Therefore in the main text we discuss the possibility of three scenarios to explain the whole data set under different culture conditions.

Supplementary Note 2 Radiogenic and stable Sr isotope analyses of coccoliths produced under the various experimental treatments are shown in Supplementary Fig. 1. Coccolith $^{87}Sr/^{86}Sr$ ratios are consistent with that of the culture medium and invariant to treatment condition, with an average value of 0.708015 ± 8. The average $\delta^{88/86}Sr$ value in coccoliths is -0.021 ± 0.044 ‰ (2 σ, n = 6), which is about 0.14‰ lower than that in the culture medium ($\delta^{88/86}Sr$ = 0.114). There is no resolvable variation for stable strontium isotopic compositions in the coccoliths from the various treatments. Stevenson, et al. [1] showed a temperature dependent growth effect can impact $\delta^{88/86}Sr$ of coccolith calcite within the species *E. huxleyi*, *Coccolithus pelagicus spp. braarudii* and *Gephyrocapsa oceanica*. In our experiment, there is no resolvable differences in coccolith $\delta^{88/86}Sr$ amongst treatments, which supports our conclusion that coccolithophore growth rate was constant across pH treatments ranging from 8.03 to 8.34, the growth rate of *O. neapolitana* does not change enough to fractionate $\delta^{88/86}Sr$.

Supplementary Note 3 Seawater carbonate chemistry throughout the experiment is shown in Supplementary Fig. 2. Temperature and salinity are nearly identical in all pCO_2 treatments, and average 24.9 ± 0.3 °C and 35.6 ± 0.2 psu, respectively (Supplementary Fig. 1(a) and (b)). Seawater DIC and total alkalinity range from 2410 to 2750 µmol kg^{-1} and 3000 to 3350 µmol kg^{-1} (Supplementary Fig. 1(c) and (d)). Calculated seawater HCO_3^-, CO_3^{2-}, $CO_{2(aq)}$ and pCO_2 concentrations are in ranges of 1890 to 2360 µmol kg^{-1}, 300 to 550 µmol kg^{-1}, 6 to 16 µmol kg^{-1} and 210 to 550 µatm, respectively (Supplementary Fig. 2 (e) to (h)).

Supplementary Figures

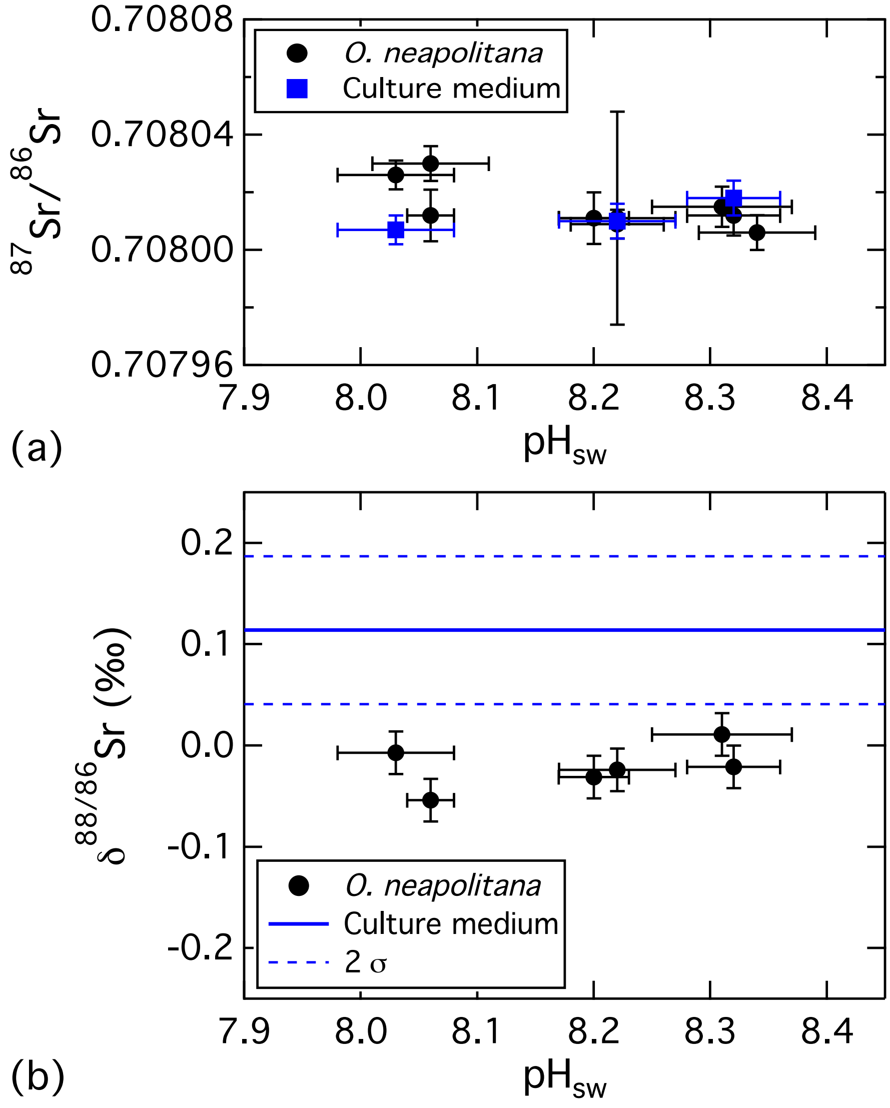

Supplementary Fig. 1 ^{87}Sr/^{86}Sr and $\delta^{88/86}$Sr as a function of ambient seawater pH. (a) ^{87}Sr/^{86}Sr and (b) $\delta^{88/86}$Sr results of coccoliths (solid black circles) and culture water medium (solid blue squares; blue line is average δ^{11}B across treatments). Error bars are 2 SD for y-axis and 1 SD for x-axis

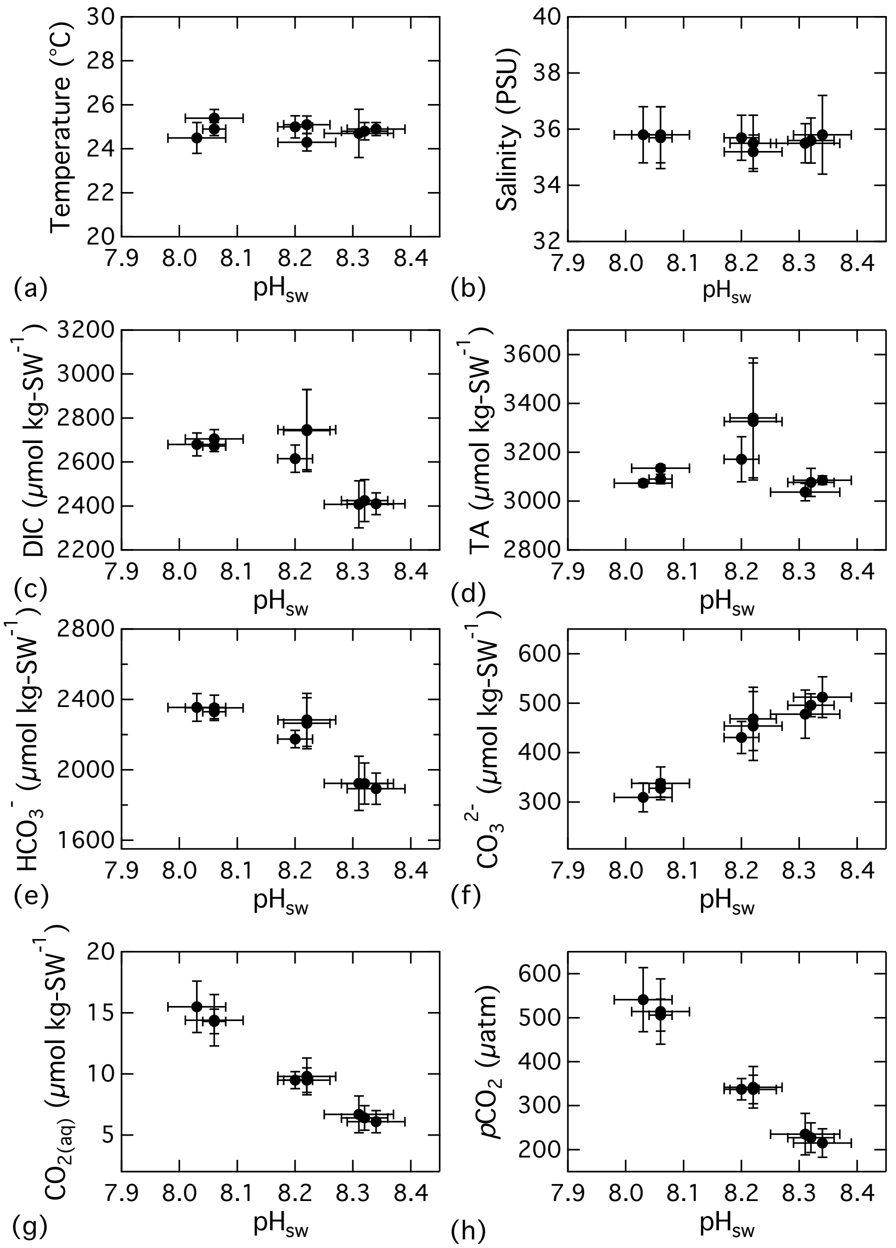

Supplementary Fig. 2 Carbonate chemistry of experimental treatments. Temperature (a), salinity (b), DIC (c) and TA (d) were measured for all treatments. Seawater pH, HCO_3^- (e), CO_3^{2-} (f), $CO_{2(aq)}$ (g) and pCO_2 (h) were calculated from the measured parameters. Error bars are 1 SD for both axis.

Supplementary Table 1 Measured and calculated parameters of the experimental seawater treatments.

	**Measured**										**Calculated**										
**pCO_2 level (replicate tank)**	**Temp (°C)**	**SD**	**Sal (PSU)**	**SD**	**TA (µmol/ kg-sw)**	**SD**	**DIC (µmol/ kg-sw)**	**SD**	**pH**	**SD**	**pH**	**SD**	**HCO_3 (µmol/ kg-sw)**	**SD**	**CO_3^{2-} (µmol/ kg-sw)**	**SD**	**CO_2 (µmol/ kg-sw)**	**SD**	**pCO_2** (µatm)	**SD**	**$\delta^{13}C_{DIC}$ (‰, VPDB)**
**Low (1)**	24.8	0.4	35.6	0.8	3077.2	57.5	2424.9	95.5	8.31	0.20	8.32	0.04	1922.5	117.3	495.9	23.1	6.4	1.0	227.3	33.9	-28.98
**Low (2)**	24.9	0.3	35.8	1.4	3085.7	18.2	2410.6	49.7	8.30	0.21	8.34	0.05	1892.3	89.0	512.3	41.2	6.1	0.9	215.4	32.4	-28.98
**Low (3)**	24.7	1.1	35.5	0.7	3037.1	35.4	2407.4	107.1	8.29	0.23	8.31	0.06	1922.9	154.2	477.8	48.7	6.7	1.5	235.4	47.4	-28.98
**Middle (1)**	24.3	0.4	35.2	0.6	3325.8	239.2	2747.5	182.8	8.20	0.20	8.22	0.05	2283.7	150.7	454.0	69.7	9.8	1.5	341.9	47.4	-6.29
**Middle (2)**	25.1	0.4	35.5	1.0	3340.5	245.4	2743.2	186.6	8.21	0.22	8.22	0.04	2265.4	145.2	468.3	64.2	9.5	1.0	337.2	32.3	-6.29
**Middle (3)**	25.0	0.5	35.7	0.8	3171.5	92.1	2615.2	62.1	8.19	0.20	8.20	0.03	2175.1	50.0	430.6	32.5	9.5	0.7	337.5	24.4	-6.29
**High (1)**	24.5	0.7	35.8	1.0	3073.6	14.2	2680.0	52.5	8.02	0.15	8.03	0.05	2355.1	78.8	309.5	29.1	15.5	2.1	541.4	72.9	-14.42
**High (2)**	25.4	0.4	35.8	1.0	3134.9	12.7	2705.1	41.9	8.03	0.22	8.06	0.05	2352.7	72.4	338.0	33.1	14.4	2.1	514.4	74.4	-14.42
**High (3)**	24.9	0.3	35.7	1.1	3090.7	19.2	2672.7	25.4	8.03	0.18	8.06	0.02	2330.5	39.0	327.8	17.7	14.3	1.0	506.3	36.3	-14.42

Supplementary References

1 Stevenson, E. I. *et al.* Controls on stable strontium isotope fractionation in coccolithophores with implications for the marine Sr cycle. *Geochim. Cosmochim. Acta* **128**, 225-235, doi:10.1016/j.gca.2013.11.043 (2014).
